# Development of a new treatment for osteoporotic vertebral fractures using adipose-derived stem cell spheroids

**DOI:** 10.1302/2046-3758.1410.BJR-2025-0092.R1

**Published:** 2025-10-28

**Authors:** Yuta Sawada, Shinji Takahashi, Kumi Orita, Akito Yabu, Masayoshi Iwamae, Yuki Okamura, Yuto Kobayashi, Hiroshi Taniwaki, Hiroaki Nakamura, Hidetomi Terai

**Affiliations:** 1 Department of Orthopedics, Osaka Metropolitan University Graduate School of Medicine, Osaka, Japan

**Keywords:** Osteoporotic vertebral fractures, Adipose-derived stem cells, Osteogenic spheroid, β-tricalcium phosphate, Bone regeneration, Paracrine effect, adipose-derived stem cells, Rats, bone formation, vertebrae, Osteoporotic bone, apoptosis, vertebral fractures

## Abstract

**Aims:**

Osteoporotic vertebral fractures substantially contribute to disability and often require surgical intervention. However, some challenges, such as implant failure and suboptimal bone regeneration, limit current treatments. Adipose-derived stem cells are promising for regenerative therapy because they are easily obtained, highly proliferative, and resistant to osteoporosis-related symptoms. This study aimed to evaluate the combined effects of osteogenic adipose-derived stem cell spheroids and β-tricalcium phosphate on vertebral bone regeneration in a rat osteoporotic vertebral fracture model.

**Methods:**

Osteoporosis was induced in 33 rats (11 per group) by ovariectomy, and defects were created in the L4 and L5 vertebrae. Adipose-derived stem cells were spheroidized and mixed with β-tricalcium phosphate scaffolds. Groups included osteogenic spheroids, undifferentiated spheroids, and β-tricalcium phosphate alone. Bone regeneration was assessed using micro-CT, histology, and biomechanical testing at four and eight weeks. Further in vitro analyses were conducted.

**Results:**

The osteogenic spheroid group showed significantly higher bone mass, fusion score, and mechanical strength than the control group did. Histological analysis revealed enhanced new bone formation and β-tricalcium phosphate integration. Gene expression analysis revealed osteogenic marker (alkaline phosphatase (*ALP*), osteocalcin (*OCN*), and runt-related transcription factor 2 (*Runx2*)) and regenerative factor (bone morphogenetic protein 7 (*BMP-7*), insulin-like growth factor 1 (*IGF-1*), hepatocyte growth factor *1* (*HGF-1*), and octamer-binding transcription factor*4* (*Oct4*) upregulation, along with reduced apoptosis. Further, adipose-derived stem cell survival was confirmed at the repair site. These results indicate that adipose-derived stem cells contribute to both paracrine and direct osteogenesis.

**Conclusion:**

Combining osteogenic adipose-derived stem cell spheroids with β-tricalcium phosphate scaffolds effectively promotes vertebral bone regeneration in osteoporotic vertebral fracture. This approach is a promising strategy for improving osteoporotic fracture repair with potential clinical applications.

Cite this article: *Bone Joint Res* 2025;14(10):915–926.

## Article focus

- Osteoporotic vertebral fractures (OVFs) contribute to disability and often require surgical intervention. However, complications such as implant failure, recurrent fractures, and nonunion remain challenges.
- Adipose-derived stem cells (ADSCs) possess regenerative potential, with spheroidization enhancing their repair ability and osteogenic differentiation further optimizing bone repair.
- This study investigates the combined effects of osteogenic ADSC spheroids and β-tricalcium phosphate (β-TCP) in promoting vertebral bone regeneration using a rat OVF model.

## Key messages

- Osteogenic ADSC spheroids combined with β-TCP significantly enhance bone formation, as demonstrated by micro-CT analysis, histological evaluation, and biomechanical testing in vivo and in vitro.
- In vitro experiments indicate that osteogenic ADSC spheroids contribute to bone regeneration through two key mechanisms: 1) direct differentiation of osteogenic ADSC spheroids and 2) paracrine effects of bone formation mediated by growth factor secretion.
- This approach provides a promising and clinically applicable regenerative therapy for OVFs, potentially improving early bone fusion and reducing complications associated with conventional treatments.

## Strengths and limitations

- This study utilizes a validated rat OVF model and introduces a novel bone fusion scoring system, ensuring objective and reproducible assessment of bone regeneration.
- The ADSC spheroidization technique is simple and scalable, making it feasible for future clinical applications in osteoporotic fracture treatment.
- The study is limited to a small-animal model, and further validation in large-animal models and long-term safety assessments are required before clinical translation.

## Introduction

Osteoporotic vertebral fractures (OVFs) are the most common fragility fractures, often leading to persistent back pain, restricted activities of daily living, and diminished quality of life, if not properly treated.^1^ Although surgery is required for refractory cases, challenges such as correction loss and implant failure persist,^2^ with no existing standardized surgical method. Therefore, improvements in surgical treatment are needed.

In osteoporotic vertebrae, the thin and fragile cortical and cancellous bone collapses, creating defects in the collapsed area even after reduction, leading to re-collapse. Bone mesenchymal stem cells (BMSCs) and adipose-derived stem cells (ADSCs) have attracted attention as regenerative therapy for refractory fractures, and many studies using animal models have been conducted.^3,4^ Although BMSCs exhibit high bone differentiation potential, their collection can be challenging in patients with osteoporosis and sparse bone tissue.^5^ Conversely, ADSCs are highly useful owing to their ease of collection under local anaesthesia,^6^ and more reliable culture potential than that of bone marrow stem cells, even in elderly patients with sparse bone marrow. Furthermore, the proliferative capacity and osteogenic differentiation of ADSCs are less affected by ageing and osteoporosis than those of BMSCs.^7^ The ideal bone graft for a fracture repair should be biocompatible, possess similar mechanical strength to bone, and promote bone formation and conduction. ADSC spheroidization has been reported to enhance tissue repair ability,^8^ with ADSC spheroid pre-differentiation improving bone repair effect.^9^ These outcomes can be achieved by inducing ADSC differentiation and spheroid formation. Conversely, undifferentiated ADSCs tend to differentiate into adipocytes when bone differentiation is not induced,^10^ and their bone regeneration potential is limited,^11^ leading to some scepticism about their efficacy. Despite this, studies using ADSCs in OVF treatment remain limited, with few studies employing animal models for OVF therapies. In this study, we used β-tricalcium phosphate (β-TCP) as a scaffold, as OVF causes structural collapse that requires reconstruction. Previous reports suggest that favourable bone regeneration can be achieved using ADSCs (as a scaffold) and β-TCP.^4^

This study aimed to verify whether combining ADSC bone differentiation spheroids and β-TCP is effective for the regeneration of a fractured vertebral body (VB) using a rat OVF model.

## Methods

### In vivo study design

**Fig. 1:**
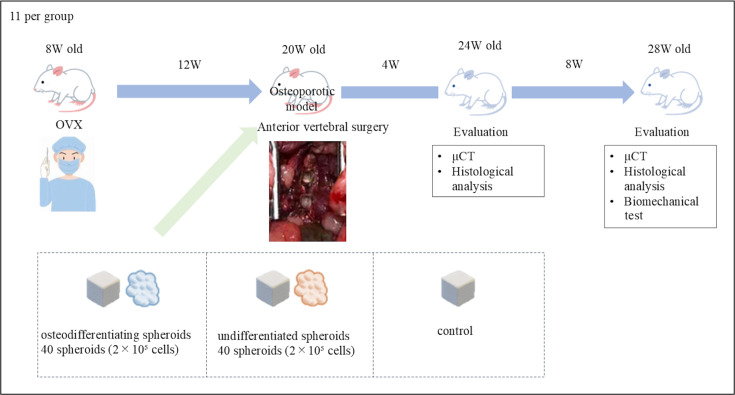
In vivo study design. Three groups were compared: the osteogenic spheroid group, in which osteogenic spheroids and β-tricalcium phosphate (β-TCP) were transplanted; the undifferentiated spheroid group, in which undifferentiated spheroids and β-TCP were transplanted; and the control group, in which only β-TCP was transplanted. Each group included 11 female Sprague-Dawley rats: ovariectomy (OVX) was performed at eight weeks, osteoporosis was confirmed at 20 weeks, and a defect was created in the vertebrae at L4 and L5 using a drill. Each group underwent transplantation. The animals were euthanized four and eight weeks after transplantation, and micro-CT, histological analysis, and mechanical evaluation were performed on the lumbar vertebrae (L4 and L5).

This study compared three groups: the osteogenic spheroid group (osteogenic spheroids + β-TCP), the undifferentiated spheroid group (undifferentiated spheroids + β-TCP), and the control group (β-TCP only). The sample size was calculated based on Cohen’s methodology,^12^ with an effect size of 0.4, assuming a one-way analysis of variance (ANOVA), a significance level of 5%, and a power of 80%. This predicted a large effect of osteogenic spheroids on bone regeneration in rat vertebral defects. Using G*Power 3.1.9.2 software,^13^ the required sample size was calculated to be 66 cases, with two vertebrae harvested per rat, thus requiring 11 rats per group. Rats were randomly assigned to groups. In the osteogenic and undifferentiated spheroid groups, ADSCs were isolated, frozen, thawed, and cultured for seven days in either osteogenic medium using the MesenCult Osteogenic Stimulatory Kit (STEMCELL Technologies, Canada) or Dulbecco’s Modified Eagle Medium (DMEM; Wako, Japan) supplemented with 10% fetal bovine serum (FBS; Wako). The spheroids were generated after 24 hours in a 96-well V-bottom plate (MS-9096U; Sumitomo Bakelite, Japan). Ovariectomy (OVX) was performed on each group of Sprague-Dawley (SD) rats (8 weeks old, female); 12 weeks later, a defect was created in the anterior L4 and L5 vertebrae using a drill, and 40 spheroids (2 × 10^5^ cells) with β-TCP were transplanted into the (hole). The animals were euthanized at four and eight weeks after transplantation, and micro-CT, histology, and mechanical evaluation were performed on the harvested lumbar vertebrae (L4, L5) (Figure 1). An ARRIVE checklist is included in the Supplementary Material to show that the ARRIVE guidelines were adhered to in this study.

### ADSC isolation

Isolation of ADSCs followed a previously described protocol.^14^ In summary, subcutaneous adipose tissue was collected from the inguinal region of eight-week-old female transgenic SD rats under anaesthesia (subcutaneous injection of ketamine 50 mg/ml and xylazine 0.2 mg/ml at a 10:3 ratio, 1 ml/kg body weight) to visualize ADSCs labelled with green fluorescent protein (GFP). These rats were of the same strain as the experimental animals, and the ADSCs were used as allogenic transplants. Harvested tissue was digested in 5 ml of type VIII collagenase (1 mg/ml in 1% bovine serum albumin/Hanks’ balanced salt solution; Life Technologies Japan, Japan) for 40 minutes at 37°C using a Bioshaker BR15 (TAITEC, Japan). Subsequently, the digested tissue was filtered through a 40 µm cell strainer (BD Falcon, Japan) and centrifuged as previously described.^14^ Cells were pelleted, suspended in lysing buffer solution (BD Biosciences, USA), and centrifuged. Pelleted cells were resuspended in an ethylenediaminetetraacetic acid (EDTA) solution, centrifuged, and mononuclear cells were collected to be used as fresh ADSCs. Following culture in DMEM (Wako) supplemented with 10% FBS (Wako), rat ADSCs between passages 4 and 6 were used in subsequent experiments.

### 3D culturing of ADSC spheroids

Following a slightly modified version of the previously described protocol, approximately 5 × 10^3^ cells in 100 μl of medium were dispensed onto the lids of 96-well V-bottom plates (MS-9096U; Sumitomo Bakelite, Japan).^14^ The ADSCs were incubated at 37°C with 5% CO₂ in DMEM containing 10% FBS (Wako) for 24 hours, and the formation of spheroids was verified under a microscope before use.

### Preparation of osteogenic differentiated ADSC spheroids

ADSCs were cultured in a bone differentiation medium using a MesenCult Osteogenic Stimulatory Kit (STEMCELL Technologies) for seven days, according to previously published methods.^15^ Spheroid formation was induced in an incubator at 37°C with 5% CO_2_ in the same medium for 24 hours, using 96-well V-bottom plates.

All in vivo studies included eight-week-old female SD rats purchased from Japan SLC (Hamamatsu, Japan). The rats were fed standard diets and housed in climate-controlled conditions. Following bilateral OVX (n = 9) or sham surgery (n = 8), as described in a previous report,^16^ rats were undisturbed for a 12-week period to establish an osteoporotic animal model. After this period, the rats were euthanized, and the proximal femur, proximal tibia, and lumbar vertebrae from L1 to L3 were harvested for bone mineral density (BMD) and micro-CT to confirm the establishment of the osteoporotic model. The L4 and L5 vertebrae were not used for osteoporosis evaluation because they were reserved for defect creation and transplantation. To minimize the number of killed animals, the same rats were used for osteoporosis confirmation and subsequent in vivo experiments. Osteoporotic bone in the OVX rats was defined as a BMD measurement > 2.5 SDs below the mean BMD of the sham-operated group, as established in a previous study.^17^

### Procedure for anterior vertebral surgery and ADSC transplantation

Based on a previously reported procedure, a defect was created in the lumbar spine of the rat using an anterior approach.^18^ Briefly, the rats were anaesthetized with ketamine/xylazine solution administered via subcutaneous injection, and the spine was exposed through a midline transperitoneal approach. After the L4 and L5 VB were identified, a defect (2 mm deep, 3 mm wide, and 4 mm long) was created in the anterior central portion of the VB using a motorized 3 mm spherical ball burr. β-TCP scaffolds were then implanted into each defect. For the osteogenic and undifferentiated spheroid groups, 40 spheroids (2 × 10^5^ cells) were dropped onto sterilized β-TCP (SUPERPORE; PENTAX, Japan) with a small amount of saline solution immediately before transplantation. The total number of cells was set at 2 × 10^5^ based on previous reports,^19^ with 5 × 10^3^ cells per spheroid, as a single spheroid diameter > 500 μm can lead to apoptosis due to insufficient oxygen, nutrients, and growth factors.^20^ The rat ADSCs within the spheroids were between passages 4 and 6.

### Micro-CT analysis

Bone regeneration within the VB defects was assessed using a micro-CT scanner (LaTheta LCT-200; Hitachi Aloka, Japan) configured at 50 kV and 0.5 mA. All rats from the three groups were anaesthetized, and scanned at zero, four, and eight weeks following ADSC implantation. As outlined previously, the reconstructed scan data were analyzed to quantify new bone volume using a 3D image processing software (ExFact VR; Nihon Visual Science Inc, Japan).^21^ The bone tissue volume located between the two pedicles of the VB was identified and defined as the region of interest (ROI). VB bone volume was defined as ROI volume. Measurements obtained at four and eight weeks were normalized to baseline data collected at zero weeks from the same animal, following established methods.^18^

### Development of a bone fusion score

**Fig. 2:**
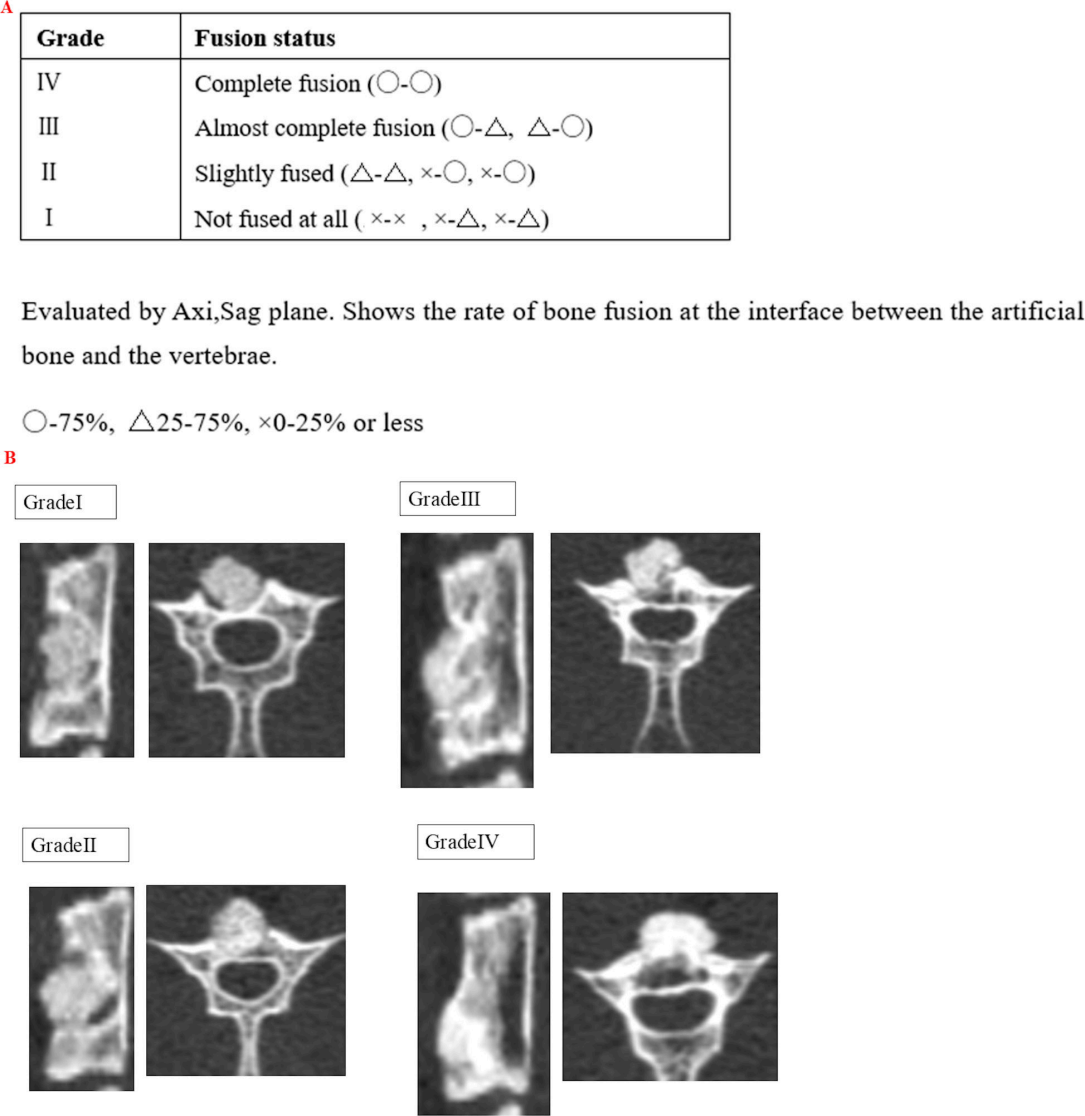
a) Typical case of a bone fusion score for the vertebral body and artificial bone in rats. b) Bone fusion score for the vertebral body and artificial bone in rats.

A bone fusion scoring system was developed to evaluate the VB and artificial bone in rats. Specifically, micro-CT imaging was performed at four and eight weeks postoperatively, and evaluations were conducted in the axial and sagittal planes at the centre of the vertebra using a DICOM Viewer. The degree of bone fusion between the VB and artificial bone was categorized into three levels for each plane, and the results were integrated to classify the outcomes into four grades (Figure 2a). Figure 2b illustrates a typical case of a bone fusion score. Training on grade determination was conducted using actual cases, after which three evaluators (YS, ST, HTa) independently graded all cases. The grading process was repeated after one week to assess consistency. Intra- and inter-rater reliability were evaluated using the intraclass correlation coefficient (ICC).

### Biomechanical testing

Seven rats from each group were killed at eight weeks post-surgery. Compression tests of the fractured vertebrae were performed for each group using a compression testing machine (EZ Graph; Shimadzu, Japan), as previously described.^22^ The strength of the fractured vertebrae was evaluated by measuring the maximum load.

### Histological and IHC analysis

Specimens were collected from the lumbar spine at four or eight weeks post-implantation, fixed overnight in 4% paraformaldehyde (Wako), and decalcified in 0.5 M EDTA for four weeks before embedding in paraffin. Tissues were sagittally sectioned (4 µm), placed onto slides, deparaffinized with xylene, and dehydrated using ethanol. Haematoxylin and eosin (H&E) and Masson trichrome staining were applied to evaluate tissue formation. Immunohistochemical (IHC) staining was conducted to assess the distribution of surviving ADSCs via GFP labelling. In addition, IHC was performed to evaluate the expression and localization of key factors involved in bone formation and regeneration, including alkaline phosphatase (ALP), insulin-like growth factor-1 (IGF-1), and osteocalcin (OCN) in bone tissue. Paraffin-embedded sections were deparaffinized and subjected to antigen retrieval using Target Retrieval Solution 10 (Dako, USA). Endogenous peroxidase activity was blocked by 3% hydrogen peroxide, and nonspecific binding was inhibited using goat serum (Nichirei Biosciences Inc, Japan). For GFP detection, sections were incubated for two hours at 20°C with an anti-GFP polyclonal antibody (1:1,000; MBL, Japan). For bone-related markers, sections were incubated for two hours at 20°C with the following primary antibodies: anti-ALP monoclonal antibody (1:100; Santa Cruz Biotechnology, USA; sc-365765), anti-IGF-1 polyclonal antibody (1:100; Proteintech, USA; 28530-1-AP), and anti-OCN monoclonal antibody (1:100; Abcam, UK; ab93876). All sections were then incubated with a peroxidase-conjugated secondary antibody (Histofine Simple Stain, Nichirei Biosciences Inc) for one hour. After washing with phosphate-buffered saline, immunoreactivity was visualized using 3,3'-diaminobenzidine (DAB; Histofine Simple DAB solution, Nichirei Biosciences Inc). The DAB reaction time was three minutes for GFP staining and one to two minutes for ALP, IGF-1, and OCN staining. Sections were counterstained with haematoxylin and observed under a BX53 microscope (Olympus, Japan).

### Apoptosis assay

Apoptosis in four groups of ADSCs was assessed using the Caspase-Glo 3/7 3D assay kit (#G8090; Promega, Germany) following an established protocol.^19^ Briefly, either 2D ADSCs (undifferentiated or osteodifferentiated) at 2 × 10^5^ cells per well or ADSC spheroids (undifferentiated or osteodifferentiated) at 2 × 10^5^ cells per 20 spheroids per well were plated in white 96-well plates (#136101; Thermo Fisher Scientific, USA) with 100 µl DMEM containing 1 µg/ml lipopolysaccharide (LPS), followed by incubation. After adding 100 µl of Caspase-Glo 3/7 reagent and incubating further, luminescence values were recorded at 12, 24, and 48 hours post-LPS exposure with a Varioskan LUX multimode microplate reader (Thermo Fisher Scientific).

### qRT-PCR analysis

qRT-PCR was used to evaluate gene expression in the four ADSC groups (both undifferentiated or osteodifferentiated, 2D ADSCs or ADSC spheroids). Total RNA were extracted from 2 × 10^5^ cultured cells (20 spheroids, as described above) using the RNeasy Mini Kit (QIAGEN, Germany). Subsequently, complementary DNA (cDNA) synthesis was performed with the High-Capacity RNA-to-cDNA Kit (Thermo Fisher Scientific). All reactions used the 7500 Fast Real-time PCR system, employing SYBR green (SYBR Premix Ex Taq; Takara, Japan) following an established protocol.^14^ The expression of glyceraldehyde-3-phosphate dehydrogenase (*GAPDH*), bone morphogenetic protein-2 (*BMP-2*), bone morphogenetic protein-7 (*BMP-7*), *IGF-1*, hepatocyte growth factor-1 (*HGF-1*), octamer-binding transcription factor 4 (*Oct4*), runt-related transcription factor 2 (*Runx2*), *ALP*, collagen type 1 A (*Col1a*), and *OCN* were analyzed. *GAPDH* was used as the reference gene (Supplementary Table i). Relative gene expression was calculated by evaluating threshold cycle (Ct) values and was normalized to undifferentiated 2D ADSCs, using the 2⁻^ΔΔCT^ method.^23^

### Statistical analysis

Results are presented as means with 95% CIs. Differences between groups were evaluated using ANOVA, followed by a Tukey post-hoc test for multiple comparisons. An independent-samples *t*-test was used for comparisons of BMD between the OVX and sham groups. The bone fusion scores for rat vertebrae and artificial bones were assessed using ICC, categorized as excellent (0.75 to 1.00), good (0.60 to 0.74), fair (0.40 to 0.59), and poor (< 0.40).^24^ Statistical significance was defined as p < 0.05, with all p-values being two-sided. Statistical analyses were conducted using R version 4.2.1 (The R Foundation for Statistical Computing, Austria) through the EZR graphical user interface (Saitama Medical Centre, Jichi Medical University, Japan). We have added this information to the revised manuscript.

## Results

### Establishment of a rat osteoporosis model

The BMD results in micro-CT for the OVX and sham groups are shown in Table I. In all regions, including the lumbar spine, the mean BMD of the OVX group was > 2.5 SD below the mean BMD of the sham group, confirming osteoporosis.

**Table I.** Results of bone mineral density (BMD) in micro-CT for the ovariectomy (OVX) and sham groups.

Mean BMD, mg/cm³ (SD)	Sham group (n = 8)	OVX group (n = 9)	p-value*
Proximal femur	757.3 (23.0)	624.3 (67.3)	< 0.001
Proximal tibia	638.2 (15.2)	498.5 (40.1)	< 0.001
L1-3	514.5 (11.9)	435.1 (25.0)	< 0.001

* Independent-samples *t*-test.

### Assessment of bone defect regeneration in vivo using micro-CT

**Fig. 3:**
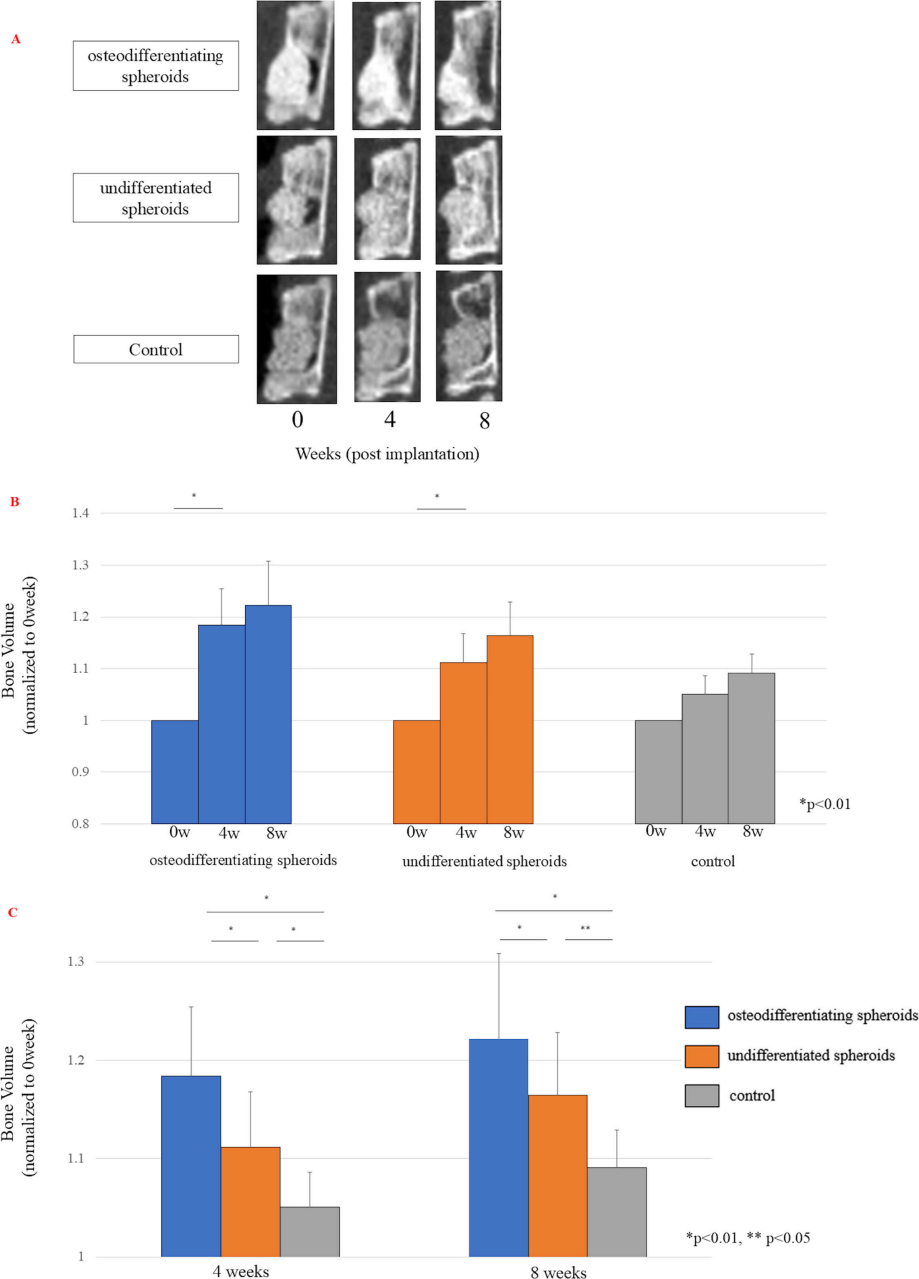
a) Serial CT images of representative cases in each group. b) Comparison of the bone volume of the vertebral body (VB) at different timepoints. Data are presented as the mean (SD). p-values were determined using a paired *t*-test (0 and 4 weeks: n = 22 per group, 8 weeks: n = 18 per group). c) Bone volume comparison of the VB among the three groups at each timepoint. Data are presented as the mean (SD). p-values were determined by one-way analysis of variance with a post hoc Tukey test (4 weeks: n = 22 per group, 8 weeks: n = 18 per group).

The effects of ADSC osteogenic spheroids, undifferentiated spheroids, and controls on lumbar bone defects were monitored and quantitatively analyzed by serial quantitative CT at zero, four, and eight weeks after implantation. The sagittal images of the representative mid-VDs are shown in Figure 3a. Treatment with ADSC osteogenic and undifferentiated spheroids incurred a significant increase in bone volume, especially in the early stages of bone repair, up to four weeks after transplantation (Figure 3b). Consequently, at both four and eight weeks post-transplantation, the bone volume in the osteogenic and undifferentiated spheroid groups was higher than that in the control group, with the osteogenic spheroid group having the greatest increase in bone volume (Figure 3c).

### Bone fusion score analysis for rat vertebrae and artificial bones

**Fig. 4:**
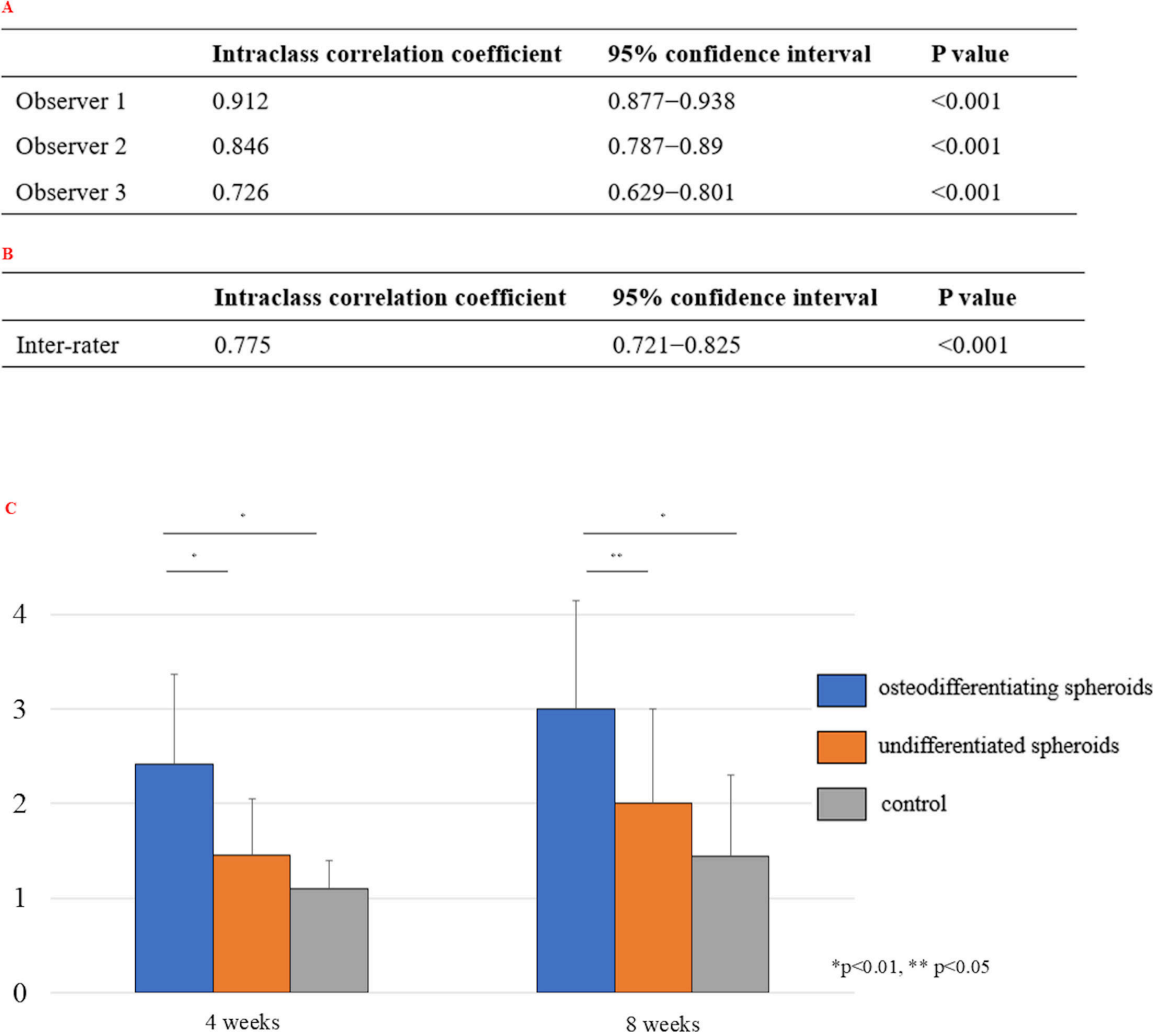
a) Intrarater reliability for bone fusion score for rat vertebrae and artificial bones. b) Inter-rater reliability for bone fusion score for rat vertebrae and artificial bones. c) Comparison of bone fusion scores for rat vertebrae and artificial bones. Data are presented as the mean (SD). p-values were determined by one-way analysis of variance with a post hoc Tukey test (4 weeks: n = 22 per group, 8 weeks: n = 18 per group).

Intra- and inter-rater reliability were excellent (≥ 0.75) for two observers (Y.Sawada, S.Takahashi), demonstrating the reliability of the bone fusion score for rat vertebrae and artificial bones (Figures 4a and 4b). At both four and eight weeks post-transplantation, the osteogenic and undifferentiated spheroid groups had higher bone fusion scores than the control group, with the highest scores observed in the osteogenic spheroid group (Figure 4c).

### Biomechanical analysis

**Fig. 5:**
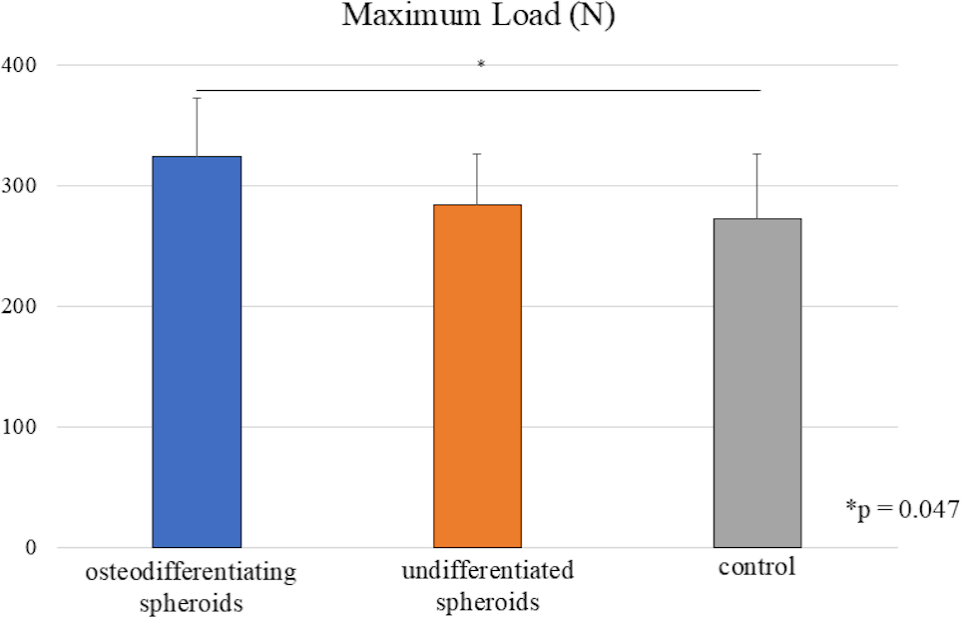
Comparison of maximum load in compression test. Data are presented as the mean (SD). p-values were determined by one-way analysis of variance with a post hoc Tukey test (osteodifferentiating spheroids: n = 12, undifferentiated spheroids: n = 10, control: n = 13).

Biomechanical testing data were excluded in cases where specimens were physically damaged during harvesting, as this rendered the samples unsuitable for accurate evaluation. This exclusion was based solely on technical reasons and not due to any biological failure or treatment-related event. The maximum load applied to the fractured vertebrae was significantly higher in the osteogenic spheroid group than in the control group (Figure 5). These results reflect a strong correlation between the mechanical function and fracture healing progress.

### Assessment of histology and IHC in vivo

**Fig. 6:**
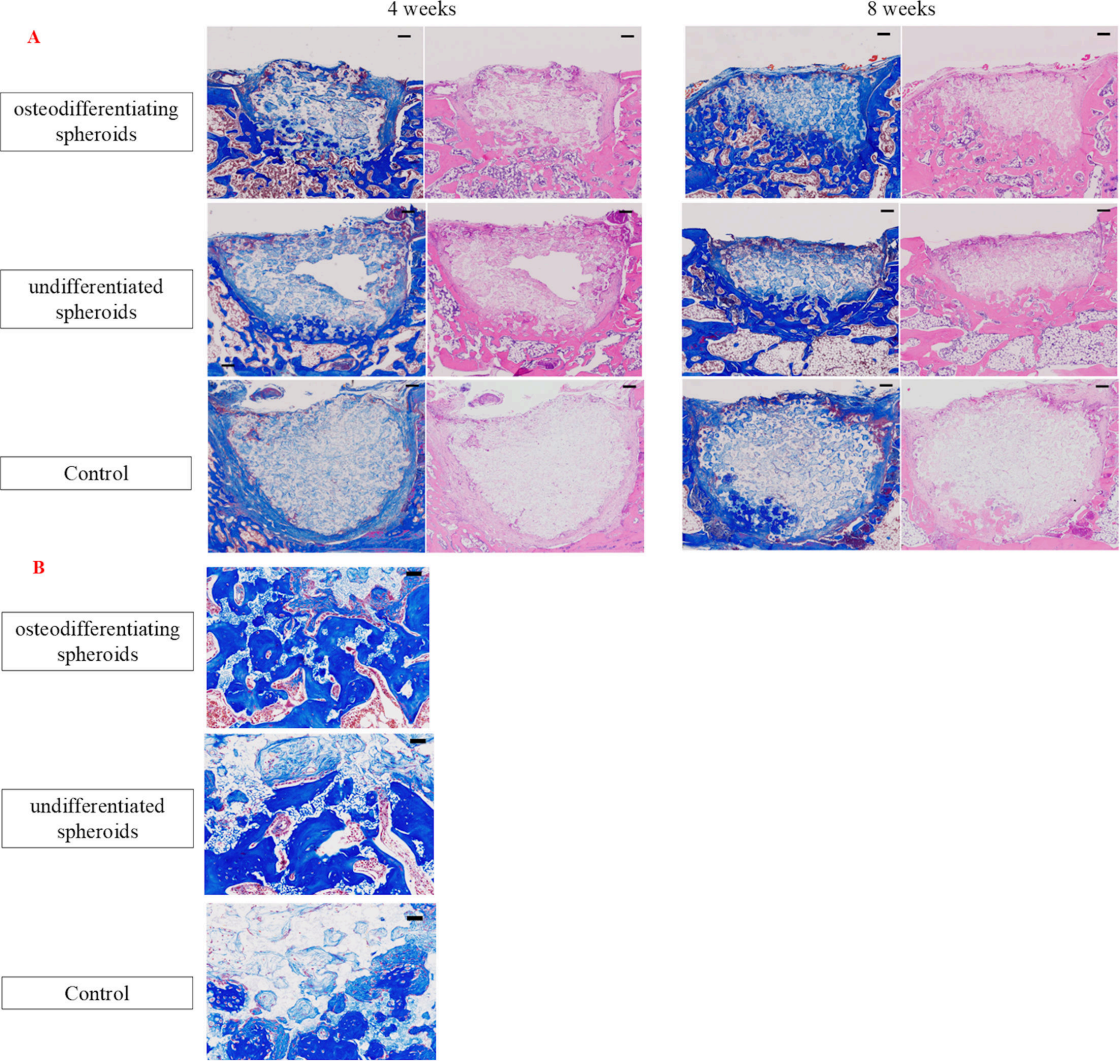
a) Representative tissue sections of the sagittal plane of the vertebral body in each group. The left side of each period shows Masson’s trichrome staining, and the right side shows haematoxylin and eosin (HE) staining. Scale bars = 200 μm. b) Magnified views of Masson’s trichrome-stained sections at eight weeks post-transplantation. Scale bars = 50 μm.

**Fig. 7:**
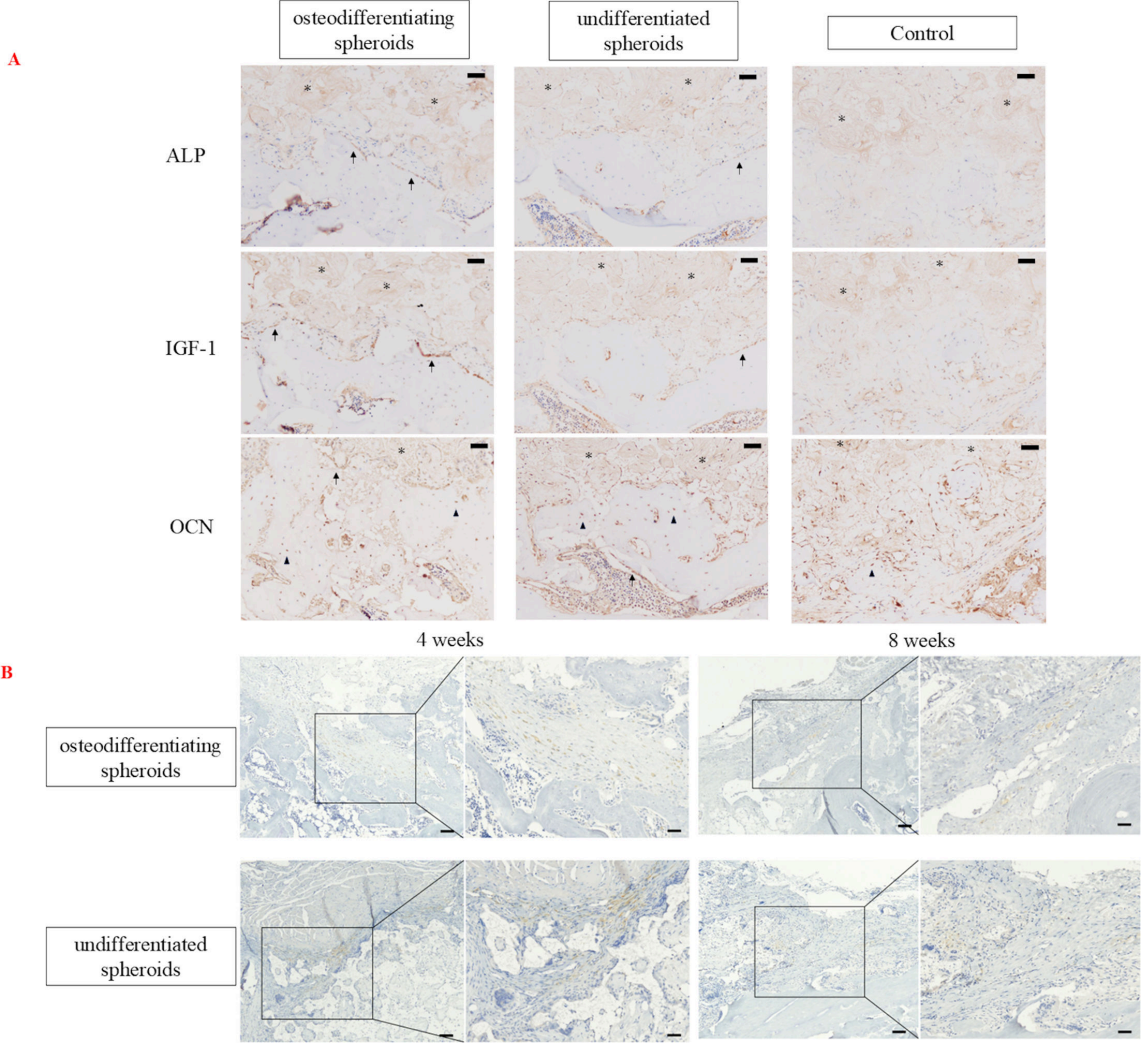
a) Representative immunohistochemistry of alkaline phosphatase (ALP), insulin-like growth factor 1 (IGF-1), and osteocalcin (OCN) in bone tissue sections at eight weeks post-transplantation. Osteoblasts are indicated by black arrows, and osteocytes are indicated by arrowheads. β-TCP is indicated by asterisks. Scale bars = 50 μm. b) Representative immunohistochemistry of green fluorescent protein (GFP) in each group over four and eight weeks. The immunohistochemical staining was performed using an anti-GFP antibody, and brown colouration indicates positive staining for GFP, reflecting the presence of transplanted adipose-derived stem cells. Scale bars = 100 and 50 μm in the left and right sides, respectively, at each period.

The bone regeneration quality was evaluated through HE and Masson’s trichrome staining at four and eight weeks post-transplantation (Figure 6a). Bone regeneration in the defect area was significantly higher in the ADSC osteogenic spheroid group than in the ADSC undifferentiated spheroid and control groups. A representative slide is shown in Figure 6a. In the ADSC osteogenic and undifferentiated spheroid groups, β-TCP was replaced by bone as time progressed, with more pronounced replacement observed in the osteogenic spheroid group. In the control group, scar tissue was mainly observed around the bone defect area. Histological evaluation of bone regeneration also showed that the ADSC osteogenic spheroid group had greater bone regeneration than the ADSC undifferentiated spheroid and control groups. In Figure 6b, especially in the osteogenic spheroid group, a layer of flattened to cuboidal mononuclear cells was observed lining the surface of the newly formed bone around the β-TCP scaffold. These cells displayed morphological characteristics consistent with osteoblast-like cells, suggesting increased osteoblastic activity compared to the other groups. In addition, the osteogenic group showed a markedly greater degree of new bone formation surrounding the scaffold compared to the undifferentiated and control groups. IHC analysis for ALP, IGF-1, and OCN revealed distinct patterns of expression among the groups (Figure 7a). In the ADSC osteogenic spheroid group, ALP and IGF-1 were strongly expressed in osteoblast cells along the newly formed bone surfaces, indicating active bone formation. In contrast, decreased expression was observed in the undifferentiated spheroid and control groups. OCN expression was detected in both the ADSC osteogenic and undifferentiated spheroid groups, primarily in osteocytes embedded in the regenerated bone matrix and in a few mature osteoblasts.

IHC staining for GFP was conducted at four and eight weeks post-transplantation to investigate the localization of the surviving ADSCs (Figure 7b). At both timepoints, GFP-positive cells were observed in the immature bone-like tissue surrounding the artificial bone in the osteogenic and undifferentiated spheroid groups.

### Apoptosis assay in the four ADSC groups in vitro

**Fig. 8:**
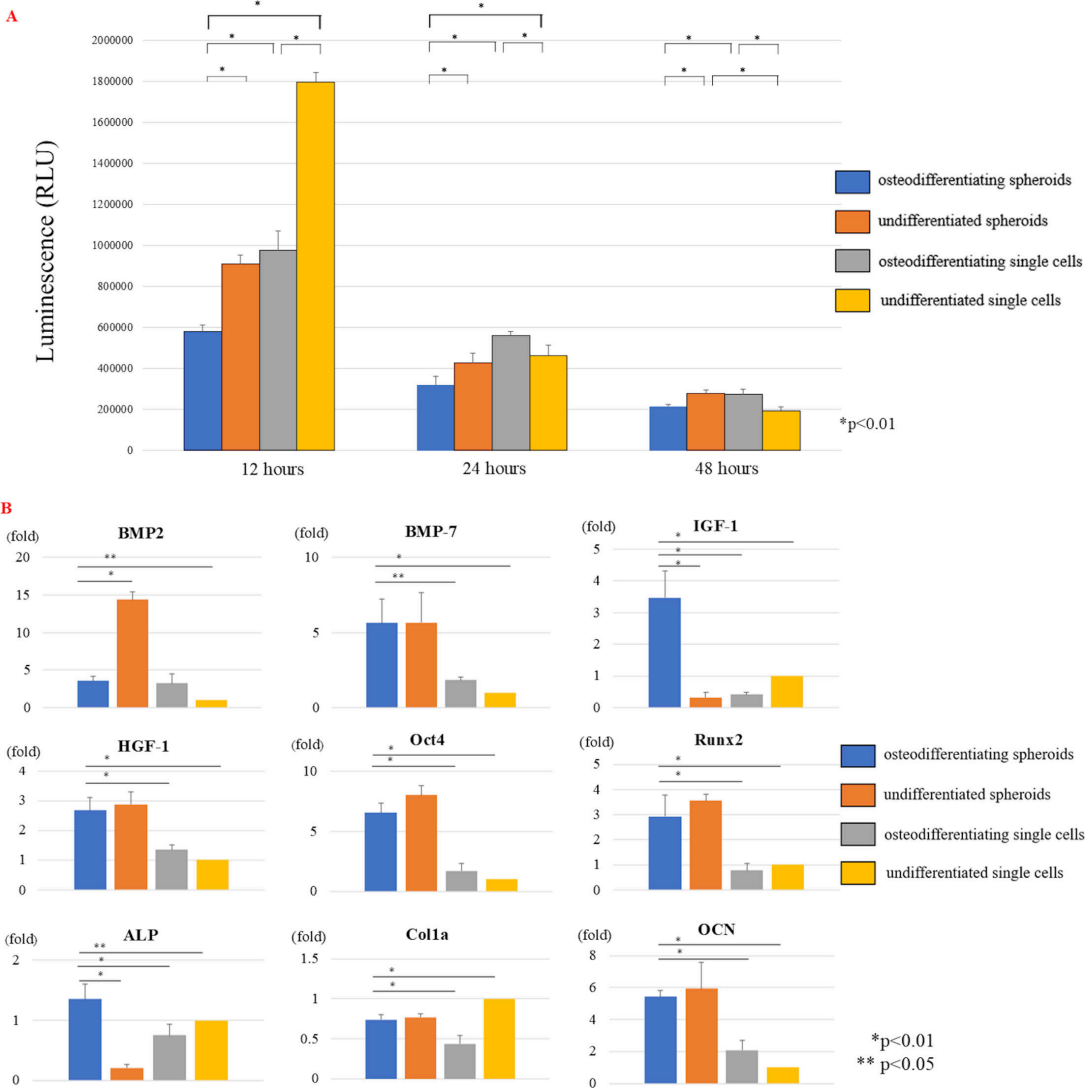
a) Apoptosis assay in the four adipose-derived stem cell (ADSC) groups. In vitro apoptosis assay of the four ADSC groups. The luminescence intensity, indicating apoptosis in each ADSC cell, was compared. Data are presented as the mean (SD). p-values were determined by one-way analysis of variance (ANOVA) with a post hoc Tukey test (n = 5 per group). b) Comparison of osteodifferentiating spheroids of each group. In vitro gene expression analysis of osteodifferentiating spheroids in each group. The relative gene expression normalized to glyceraldehyde 3-phosphate dehydrogenase (GAPDH) was compared. Data are presented as the mean (SD). p-values were determined by one-way ANOVA with a post hoc Tukey test (octamer-binding transcription factor4 (Oct4), alkaline phosphatase (ALP), and osteocalcin (OCN): n = 4, others: n = 3).

Compared to ADSC-osteogenic single cells, apoptosis was significantly suppressed in osteodifferentiating ADSC spheroids at 12, 24, and 48 hours (p < 0.01; Figure 8a). Furthermore, apoptosis was also significantly suppressed (p < 0.01) in osteodifferentiating ADSC spheroids at 12, 24, and 48 hours compared with undifferentiated ADSC spheroids. Moreover, apoptosis at 12 and 24 hours was significantly suppressed in the ADSC osteogenic spheroids compared with the other three groups (p < 0.01). These results indicate that spheroidization and osteogenic differentiation lead to higher cell viability than single-cell morphology, with the effect being more pronounced in osteogenic spheroids.

### Gene expression of the four ADSC groups in vitro

The expression of *ALP* and *IGF-1* was significantly higher in the osteodifferentiating ADSC spheroid group than in the other groups (Figure 8b). Moreover, the expression of the osteogenic markers, *Runx2* and *OCN*, was significantly higher in ADSC osteodifferentiating spheroids than in osteodifferentiating or undifferentiated single cells. Furthermore, *BMP7*, *HGF-1*, and *Oct4* expressions were significantly higher in ADSC osteodifferentiating spheroids than in osteodifferentiating or undifferentiated single cells. In addition, *BMP2* expression was significantly greater in the ADSC undifferentiated spheroid group than in the other three groups.

## Discussion

This study demonstrated that ADSC bone differentiation spheroids have a strong bone repair effect in a rat OVF model. In general, both in vivo and in vitro results highlight the spheroids’ significant regenerative effect.

Although previous studies have shown the bone repair effects of MSC-derived bone differentiation spheroids,^9^ this is the first study to demonstrate the effectiveness of spheroidizing ADSCs after bone differentiation. Inducing osteogenesis after spheroidization is often challenging using conventional methods of adding external factors, such as osteogenic culture medium, owing to diffusion limitations.^25^ These methods often require special techniques, such as uniformly mixing BMP-2-coated fibres into the spheroid.^9^ We successfully produced ADSC bone spheroids using a simple method of spheroidizing ADSCs after bone differentiation by employing bone differentiation medium and the hanging-drop method.^26^ Another novel aspect of this study lies in its establishment of a bone fusion score for rat VBs and artificial bones in a rat vertebral defect model. The scoring system was developed by focusing on the relationship between the degree of bone fusion at each vertebra cross-section and the amount of new bone formation. The ICC evaluation showed high reproducibility and reliability of the bone fusion score, and its effectiveness was confirmed using micro-CT, enabling objective and quantitative evaluation. Furthermore, GFP-labelled ADSCs improved understanding of the mechanism underlying spheroid therapy.

**Fig. 9:**
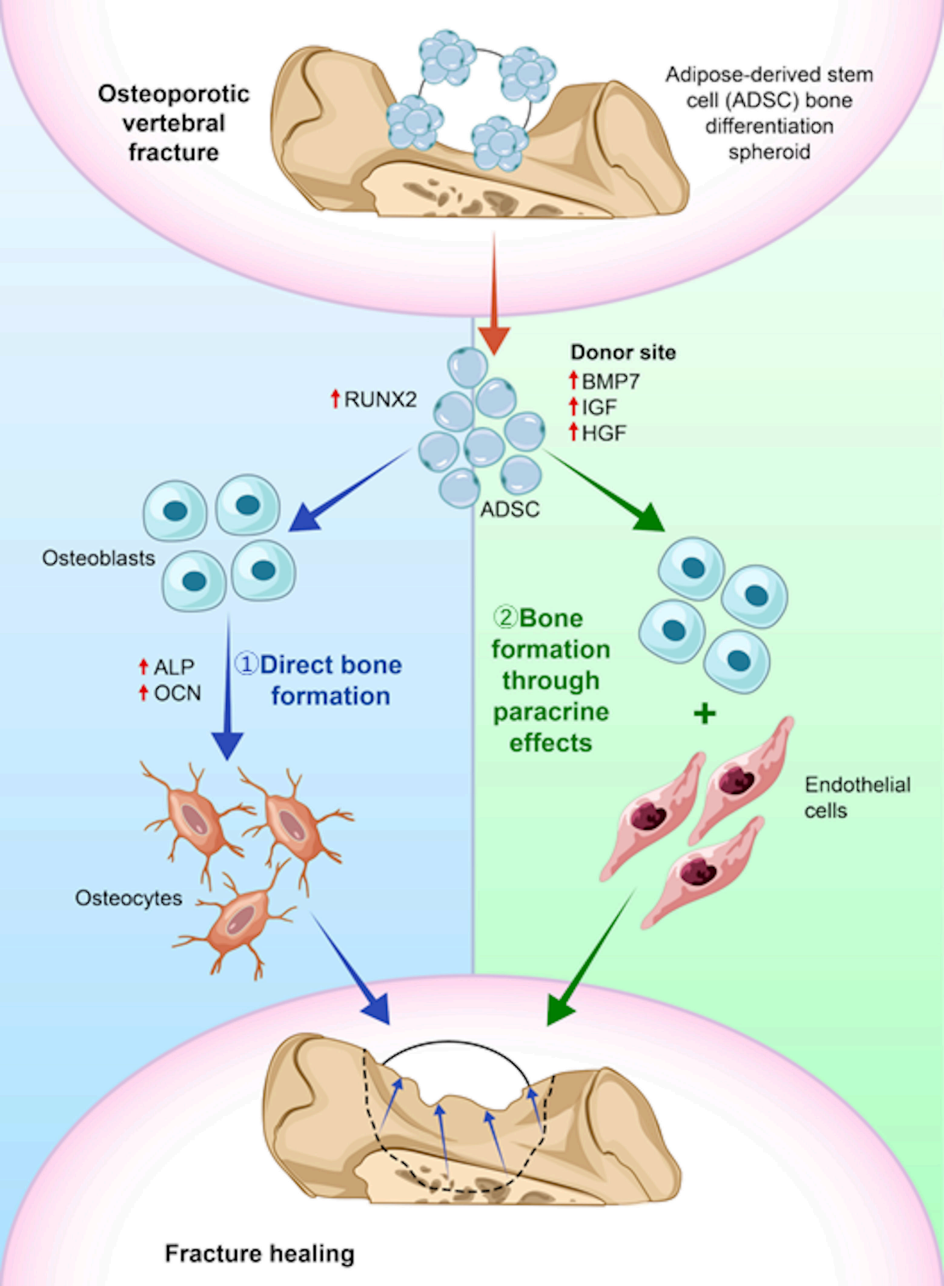
Bone regeneration mechanism of adipose-derived stem cell (ADSC) bone differentiation spheroids. 1) Direct bone formation through autodifferentiation of transplanted ADSC bone differentiation spheroids. 2) Promotion of bone formation through paracrine effects of growth factor secretion.

After transplantation of the ADSC spheroids, GFP-positive cells were observed in the immature bone-like tissue around the artificial bone in both the bone-differentiated and undifferentiated groups, suggesting that paracrine effects from ADSCs are involved in new bone formation. Previous studies have shown that osteogenic spheroids promote bone formation via paracrine effects.^12^ The current study further supports this finding, as gene expression analysis also confirmed an increase in the expression of the bone formation induction markers, *BMP-7*^27^ and *IGF-1*;^28^ tissue repair marker, *HGF-1*;^29^ and stem cell marker, *Oct4*.^30^ Specifically, *IGF-1* alone has been shown to induce osteogenesis,^31^ and a report has suggested that it works synergistically with *BMP-7* to promote osteoblast differentiation via protein kinase C and D signalling pathways.^32^ In addition, apoptosis assays demonstrated that osteogenic spheroids suppressed apoptosis more effectively and had a positive effect on bone formation, confirming that the paracrine effect of ADSC osteogenic spheroids plays a crucial role in bone formation. Moreover, the high gene expression of bone formation markers, such as *ALP, OCN*, and *Runx2*, as well as the results of apoptosis suppression, suggests that the osteogenic spheroids contribute directly to bone formation. In particular, the upregulation of *IGF-1* and *ALP* is considered to have contributed to the enhanced bone repair observed in vivo. IGF-1 promotes osteoblast proliferation and differentiation, while *ALP* plays an essential role in early matrix mineralization. *IGF-1* has been shown to act synergistically with *BMP-7* to further accelerate osteoblast differentiation. These effects may have amplified the regenerative response, especially in the osteoporotic bone environment. This was further supported by IHC staining of bone tissue sections, which demonstrated increased expression of ALP, IGF-1, and OCN in the osteogenic spheroid group, consistent with the gene expression profiles observed in vitro. Furthermore, the superiority of ADSC spheroid culture over 2D culture in promoting bone regeneration has been demonstrated in several studies.^9,19,33^ Spheroid formation has been reported to enhance cell–cell interactions, stimulate extracellular matrix (ECM) production, and increase the secretion of growth factors involved in bone formation and tissue regeneration. Although it remains unclear whether this functional advantage is specific to osteoporotic bone, previous reports have shown that osteoblast function and bone regenerative capacity are impaired in osteoporotic bone.^34^ Under pathological conditions, the paracrine effects and high cell viability associated with spheroids may play a more prominent therapeutic role. On the other hand, enhanced bone regeneration by spheroids has also been reported in healthy bone models,^19^ suggesting that the observed regenerative effects are not limited to osteoporotic bone and may be applicable to a broader range of skeletal conditions. Regarding the difficulty in detecting GFP-positive cells in the bone tissue, the signal may have been reduced by decalcification, or the physical characteristics of the bone matrix may have limited antigen detection.^35^ Based on these results, the bone regeneration mechanism by ADSC bone differentiation spheroids is hypothesized as follows: the transplanted ADSC bone differentiation spheroids produce bone formation factors (*ALP*, *OCN*, and *Runx2*) and directly contribute to bone regeneration by differentiating into osteoblasts and osteocytes. In addition, they secrete growth factors (*BMP-7*, *IGF-1*, and *HGF-1*) and stimulate host osteoblasts, thereby promoting bone formation through paracrine effects (Figure 9). The spheroids also stimulate vascular endothelial cells, which supports bone regeneration.^36^ These dual effects enable the osteogenic spheroids to achieve de novo bone formation.

This study demonstrates that ADSC bone differentiation spheroids could be a new and effective treatment for OVFs. ADSCs are easily obtained and highly proliferative even in an elderly population with osteoporosis. In addition, bone-differentiated spheroids can be created using a simple method of inducing differentiation in a bone differentiation medium, followed by spheroidization using the hanging-drop method, which holds promise for future clinical applications. Furthermore, ADSC bone differentiation spheroids are effective in the early stages of bone regeneration after transplantation, making them advantageous in clinical applications. If a treatment system for OVF using regenerative medicine that utilizes ADSCs can be established, early bone fusion can be promoted by transplanting bone-differentiated spheroids along with β-TCP after restoring the collapsed part of the VB. This approach could be used in problematic OVF situations, such as deformity healing and bone fusion failure. In the future, we plan to conduct verification of the findings using large animals.

This study had some limitations. First, the rat vertebral fracture model involved a vertebral defect, which does not perfectly replicate human vertebral fractures. Rats are quadrupedal animals, and the load applied to the spine differs from that of bipedal species such as humans. However, axial compressive forces strongly affect the rat vertebrae, which is related to the high bone density of the vertebrae.^37^ Conversely, in compression fractures, which are the most common type of vertebral fracture in humans, bone regeneration typically occurs along the existing fracture site. However, this is not the case in the rat defect model; therefore, the rat vertebral fracture model may reproduce a more severe situation. Second, although the long-term effects and safety of ADSC spheroids after transplantation remain unclear, safety evaluations using a long-term model showed no evidence of side effects, such as tumour formation or immune rejection. Third, although the paracrine effect and direct bone regeneration are suggested to play roles in the bone regeneration mechanism, further clarification of the detailed molecular mechanism is required.^38^ In addition, this study did not include a non-spheroid ADSC group in the in vivo experiments, as the superiority of spheroid culture was demonstrated in the in vitro analyses. However, this limits the ability to directly compare the in vivo effects of cell morphology. Furthermore, gene expression was evaluated in vitro, but protein-level validation was not performed, which may provide further insight into the functional mechanisms. Finally, the conditions for bone differentiation and the optimal transplantation volume of spheroids should also be addressed in the future. In particular, in this study, spheroid formation was carried out after culturing for seven days in a bone differentiation medium based on previous reports;^15^ however, extending the culture period may further enhance bone formation potential, warranting additional research to explore this possibility.

In conclusion, this study demonstrated that ADSC bone differentiation spheroids have a powerful therapeutic effect in promoting bone repair in a rat OVF model. In addition, a simple and effective method for preparing bone differentiation spheroids and a highly reliable evaluation score were established. With further research, this approach holds the potential to develop into an innovative treatment that reduces deformity healing and nonunion after OVF, thereby improving treatment outcomes and extending healthy life expectancy.

## Data Availability

The datasets generated and analyzed in the current study are not publicly available due to data protection regulations. Access to data is limited to the researchers who have obtained permission for data processing. Further inquiries can be made to the corresponding author.
